# Risk Factors for QRS-Fragmentation in Patients with STEMI Undergoing PCI

**DOI:** 10.3390/medicina61112023

**Published:** 2025-11-12

**Authors:** Florian Tinhofer, Rosana Rakhimova, Elena A. Badykova, Lukas Fiedler, Dilvin Semo, Christoph C. Kaufmann, Irina A. Lakman, Eduard F. Agletdinov, Dimitry M. Grishaev, Ksenia A. Cheremisina, Anastasia V. Baraboshkina, Lukas J. Motloch, Rudin Pistulli, Naufal S. Zagidullin

**Affiliations:** 1 3rd Medical Department with Cardiology and Intensive Care Medicine, Clinic Ottakring, 1160 Vienna, Austria; 2 Medical School, Sigmund Freud University, 1020 Vienna, Austria; 3 Medical School, Paracelsus Medical University, Freudplatz 1, 1020 Wien, Austria; 4 Department of Internal Diseases, Bashkir State Medical University, Lenin Str., 3, 450008 Ufa, Russia; 5 Department of Internal Medicine, Cardiology and Nephrology, Landesklinikum Wiener Neustadt, 2700 Wiener Neustadt, Austria; 6 Department of Medicine, Faculty of Medicine and Dentistry, Danube Private University, 3500 Krems, Austria; 7 Department of Cardiology I, Coronary and Peripheral Vascular Disease, Heart Failure, University Hospital Muenster, 48149 Muenster, Germany; 8 Institute of Economics, Finance and Business, Ufa University of Science and Technology, Validy Str. 32, 450076 Ufa, Russia; 9 «Vector-Best» RIDT JSC, 630117 Novosibirsk, Russia; 10 Department of Internal Medicine II, Salzkammergut Klinikum, OÖG, 4840 Vöcklabruck, Austria; 11 Department of Cardiology, Kepler University Hospital, Medical Faculty, Johannes Kepler University, 4020 Linz, Austria; 12 Department of Internal Medicine I, Marien Hospital Papenburg Aschendorf, 26871 Papenburg, Germany

**Keywords:** fragmented QRS, STEMI, ACS, ECG

## Abstract

*Background and Objectives*: Despite modern therapy algorithms, ST-elevation myocardial infarction (STEMI) substantially contributes to cardiovascular morbidity and mortality worldwide. Early Risk assessment is crucial to guide therapy allocation, especially in countries with limited healthcare resources. Electrocardiographic parameters such as QRS fragmentation (fQRS) evolved as an important prognostic marker. The underlying mechanisms and specific risk factors for the occurrence of fQRS in patients with STEMI undergoing PCI have not been analyzed yet. *Materials and Methods*: Between 09/2020 and 06/2021, out of 179 consecutive patients with STEMI undergoing primary percutaneous coronary intervention (pPCI), 122 patients were included in this study. The occurrence of fQRS was analyzed and correlated to clinical as well as biochemical parameters. *Results*: In this population, the fQRS pattern was present in 33.6% (n = 41) of patients. Besides gender, no statistically significant differences in baseline characteristics or comorbidities were observed between the two groups. In univariable logistic regression analysis, both glomerular filtration rate (GFR) (*p* = 0.050) and C-reactive protein (CRP) (*p* = 0.014) were significantly associated with the presence of fQRS. However, in the multivariable logistic regression model, only CRP levels on admission remained independently associated with fQRS (OR = 3.44, 95% CI: 1.95; 6.05), (*p* = 0.029). *Conclusions*: In this analysis, a correlation between fQRS and CRP levels in patients with STEMI undergoing pPCI could be demonstrated. Consequently, fQRS might serve as a marker for extensive inflammation in the context of myocardial ischemia.

## 1. Introduction

Obstructive coronary arteriosclerosis, a common manifestation of cardiovascular disease (CVD), ranks among the leading causes for morbidity and mortality worldwide. The broad spectrum of clinical presentations ranges from stable coronary artery disease to acute coronary syndrome (ACS) [1,2,3]. Various clinical scenarios fulfill the definition of ACS, whereas the management of patients with ST-segment elevation myocardial infarction (STEMI) remains challenging due to the occurrence of short-term as well as long-term complications in this population. Early risk stratification and the identification of patients at an increased risk for adverse events appear to be crucial, especially in the scope of limited healthcare resources [4]. Apart from left ventricular ejection fraction, successful PCI, and the presence of arrhythmias, specific ECG parameters such as QRS-fragmentation (fQRS) might help to identify patients who benefit from intensified follow-up [5,6].

In recent years, fQRS has evolved as an important prognostic marker in STEMI patients undergoing primary percutaneous coronary intervention (pPCI). Several outcome parameters, such as in-hospital mortality, major adverse cardiac events (MACE), and poor left ventricular function, have been shown to correlate with the presence of fQRS. Furthermore, higher levels of myocardial enzymes as well as more severe coronary lesions during angiography have been demonstrated in patients with fQRS [7,8,9]. In combination with other ECG markers such as QRS distortion, the prognostic accuracy regarding early risk stratification can be further improved [7]. Data on the relation between infarct size and fQRS are conflicting [9,10]. However, the association between all-cause mortality and fQRS has been confirmed in a meta-analysis [11]. The prediction of poor outcomes and impaired myocardial reperfusion even appears to be possible if fQRS is only present in single ECG leads [12,13]. Importantly, there exists no universally accepted consensus for the definition of fQRS as an electrocardiographic parameter. To allow objective ECG analysis and reduce intra- and inter-observer variability, machine learning algorithms for fQRS quantification have been proposed [14].

The actual mechanisms, risk factors, and causes for fQRS are not well defined yet. Degeneration of the cardiac conduction system is favored by ischemia or local fibrosis and might be increased in patients with inflammatory diseases such as rheumatoid arthritis [15,16]. Additionally, incomplete myocardial reperfusion and lower TIMI myocardial perfusion grades were linked to fQRS [17]. A strong association between the presence of fQRS and contrast-induced nephropathy could be observed in patients with ACS undergoing interventional management [15]. Finally, the concept and relevance of temporary QRS fragmentation have not been described at all. In this analysis, we want to further investigate and specify the risk factors for the occurrence of fQRS in patients with STEMI undergoing PCI.

## 2. Materials and Methods

In this prospective single-center, non-randomized study, 122 out of 179 consecutive patients undergoing coronary angiography (CA) due to STEMI were included between September 2020 and June 2021. All patients were admitted to the cardiac center of Ufa City Hospital N21 in the Russian Federation, which is capable of performing 24/7 percutaneous catheter intervention (PCI) service. The STEMI diagnosis was established and confirmed using serial 12-Lead ECG recordings according to the currently available standards of the European Society of Cardiology (ESC) Fourth Universal Definition of myocardial infarction(MI) [4,18,19]. Acute CA, and if indicated, PCI was performed on admission. In case of delayed first medical presentation, thrombolytic therapy was applied in the absence of contraindications at the discretion of the treating primary care physician. In patients presenting with clinical or electrocardiographic evidence of failed fibrinolysis—such as recurrent ST-segment elevation suggestive of re-occlusion or reinfarction or persistent ischemia—rescue PCI was performed as promptly as possible. Acute pharmacological management, including antiplatelet therapy and discharge medications, was administered in accordance with the current guidelines of the ESC [4,18].

The study was conducted in compliance with Good Clinical Practice (GCP) guidelines and the principles outlined in the Declaration of Helsinki. Ethical approval was obtained from the Ethics Committee of Bashkir State Medical University (Approval No. 1, dated 23 January 2017). Informed consent was obtained from all participants before their enrollment in the study.

**Figure 1:**
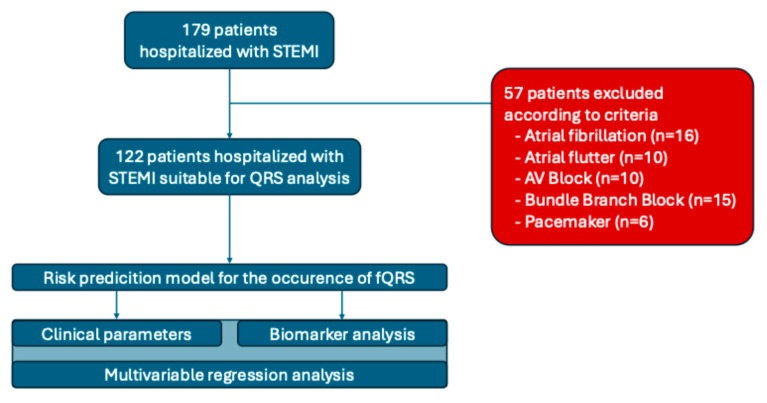
Patient population.

Within the study period, all patients presenting with STEMI at the respective institution above the age of 18 years were evaluated. Due to the following criteria 57 patients had to be excluded from further analysis: presentation >48 h from start of typical symptoms of ACS, severe valvular dysfunction defined as severe regurgitation or stenosis of one or more of the cardiac valves, dilative cardiomyopathy, permanent atrial fibrillation and/or atrial flutter, AV block II–III according to medical history and ECG, implanted pacemaker, WPW-Syndrome with apparent delta-wave, acute pulmonary embolism, active malignant disease defined as achieved tumor free survival under three years, severe chronic obstructive pulmonary disease (GOLD 2009 stage III–IV), uncontrolled bronchial asthma (according to Global Initiative for Asthma, GINA 2019), acute infectious diseases at the time of admission defined as acute pyelonephritis, community acquired pneumonia, acute bronchitis and/or flu/acute respiratory viral infection, and kidney failure defined as glomerular filtration rate (GFR) <30 mL/min1.73 m^2^ as well as pregnancy or lactation. Patient enrollment and the study design are illustrated in Figure 1.

**Figure 2:**
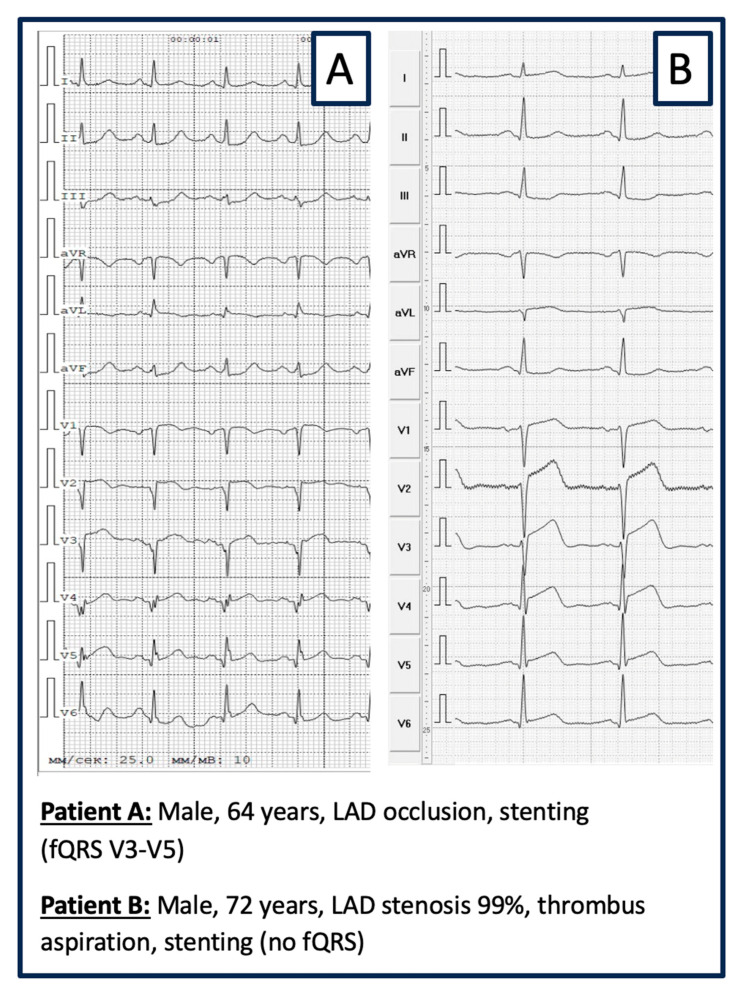
12-Lead-ECG example of STEMI with fQRS (Patient **A**) and STEMI without fQRS (Patient **B**).

- BIOMARKER ANALYSIS: Within the first hour of hospital admission and always before CA/PCI, venous blood samples were collected from all patients, centrifuged, and the resulting serum was aliquoted and stored at −80 °C for subsequent analysis. Serum concentrations of the biomarkers were measured using enzyme-linked immunosorbent assay (ELISA) kits, following the manufacturer’s instructions (Vektor-Best, Russia).
- ELECTROGRAM ANALYSIS: The ECG was recorded on ECG GE MAC 1200 ST, 12-channels: filters from 0.16 to 100 Hz; AC filter 60 Hz, ECG speed 25 mm/s, voltage 10 mm/mV. ECG evaluation regarding fQRS was performed visually by 2 experienced cardiologists blinded to the patient’s medical records and history. Inter-observer consensus regarding the presence of fQRS was mandatory. fQRS was defined as visible notching of the R- or S-wave in at least 2 contiguous leads (same coronary perfusion territory) in a routine 12-lead ECG (Figure 2) [20].

All statistical analyses were independently performed by a blinded statistical team using SPSS software (version 21) and R. Continuous variables with normal distribution are presented as mean (M) ± standard deviation (SD), while non-normally distributed variables are summarized using interquartile ranges. Group comparisons for continuous variables were conducted using the Mann–Whitney U test, selected for its superior statistical power in small, non-parametric samples. Categorical variables were compared using the Chi-square test. Although the required sample size for the study was not determined a priori, a post hoc power analysis was conducted based on the results of the logistic regression. The main criterion for the admissibility of the analysis was that the ratio of events-per-variable (in our case, 41 cases of _f_QRS) should be at least 10 times greater than the number of factors considered in the analysis models [21]. The post hoc power calculation was performed using the powerLogisticBin function from the “power mediation” package in R.

To identify risk factors of fQRS, the logistic regression was used, for which the coefficients of the regressors and their standard errors were determined by the maximum likelihood method. Regression equations were calculated in two stages: at the first stage, univariable analysis (with one factor of influence) was estimated; if the factor turned out to be statistically significant (*p* < 0.05) according to the Wald test, then it was included in the multivariable analysis. To test the robustness of the obtained estimates of the coefficients in the multivariate logistic regression model, the Hosmer–Lemeshow test for goodness-of-fit of the original and predicted data was used. Data were considered consistent if the null hypothesis was confirmed. To assess the presence of multicollinearity in the model, the VIF criterion was used, and collinearity between the factors was considered absent if VIF < 5. To ensure the admissibility of multivariate logistic regression analysis, the events-per-variable (EPV) ratio was calculated. If EPV ≥ 10, the analysis was considered appropriate. In the multivariable equation, odds ratios (OR) and their confidence interval (CI) were determined based on the calculated coefficients of the regressors at a reliability of 95%.

## 3. Results

### 3.1. Baseline and Procedural Characteristics of the Patient Cohort

This study included 122 patients selected from a cohort of 179 individuals presenting with STEMI over the period of 10 months at a tertiary cardiology center capable of performing 24/7 CA and PCI service. According to ECG parameters on presentation, the patient population was divided into a group with fQRS pattern (33.6%, n = 41) and another group without fQRS complexes (66.4%, n = 81). Besides gender, there were no statistically significant differences in baseline characteristics (Table 1) or comorbidities (Table 2) between the two groups. Additionally, no relevant differences were observed in echocardiographic (Table 3) or procedural angiographic parameters (Table 4). Continuous data are reported as median and interquartile range.

### 3.2. Risk Factors for QRS Fragmentation

To evaluate potential risk factors associated with the presence of fQRS, plasma levels of glucose, CRP, creatinine, glomerular filtration rate (GFR), urea, and aspartate aminotransferase (ASAT), Alanin–Aminotransferase (ALAT), Troponin T, and lactate dehydrogenase (LDH) were analyzed. Univariate logistic regression identified GFR, Onset-to-door time, and CRP as the only variables significantly associated with the occurrence of fQRS (Table 5). In univariate models, the categorical variables TIMI и Killip were taken as dummy. A multivariable logistic regression model incorporating these three predictors was constructed, and odds ratios (ORs) were calculated based on the model coefficients (Table 6). According to the results of logit regression, each 1-unit (mg/dL) increase in CRP unit was associated with a more than threefold increase in risk (OR = 3.22, *p* = 0.043). A sensitivity analysis was performed for the statistically significant factor “CRP” to determine whether the findings were robust. To do this, we changed the set of variables included in the logistic regression model. This is how a model was built, including only CRP and GFR, which showed statistical significance for CRP at *p* = 0.029 (OR = 3.44, CI_95%: 1.9548; 6.0503), for GFR, *p* = 0.061 (OR = 0.99, CI_95%: 0.979; 0.994). A model was also built including only CRP and Onset-to-door time variables, which showed statistical significance only for CRP at *p* = 0.024 (OR = 3.76, CI_95%: 2.09; 6.78), for Onset-to-door time, *p* = 0.078 (OR = 1.002, CI_95%: 1.001; 1.004). The results of the sensitivity analysis showed the stability of the effect of CRP level on fQRS in myocardial infarction.

**Figure 3:**
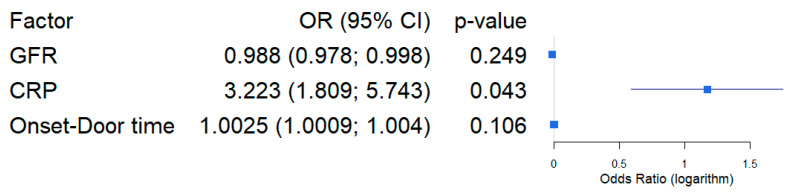
Forest plot of logarithmic scale for multivariable regression analysis of fQRS as a risk factor of STEMI patients.

For the visual presentation of OR and its confidence interval, the forest plot graphic was constructed on a logarithmic scale (Figure 3). According to the Hosmer–Lemeshow criterion (X-squared = 4.4689, df = 8, *p*-value = 0.8125), there was consistency between the calculated and original data. The VIF analysis showed that there was no collinearity between the explanatory factors in the logistic regression, since the VIF value for each of them did not exceed 5: VIF(CRP) = 1.042, VIF(GFR) = 1.048, VIF (Onset-to-door time) = 1.011. Since there are only two factors in the model, EPV = 41/2 = 20.5, which exceeds the required value of 10 to obtain reliability of the results [21].

Although the required sample size for the study was not determined a priori, a post hoc power analysis was conducted based on the results of the logistic regression. For the CRP risk factor, an OR of 3.22 was calculated. Given a significance level of 0.05, a sample size of 122, an event proportion of 0.336, and an expected fQRS probability of 0.5, the power was 0.817, exceeding the minimum required power of 80%.

## 4. Discussion

In this study, risk factors for the presence of fQRS in patients with STEMI undergoing CA and pPCI were analyzed. Various clinical, echocardiographic, procedural, and laboratory parameters were evaluated for a potential correlation with the occurrence of fQRS. Importantly, patients with comorbidities such as a previous pacemaker, AV block, or infectious diseases were excluded from this analysis to avoid the presence of confounders. By use of a multivariable regression model, only CRP levels remained independently and significantly associated with the presence of fragmented QRS complexes. Specifically, each 1-unit increase in CRP levels was associated with a 3.4391-fold increase in the odds of exhibiting fragmented QRS in these patients. To our knowledge, this is the first study to identify CRP levels as a risk marker for fQRS in a STEMI population. Linking laboratory parameters to ECG characteristics potentially allows a more comprehensive evaluation of critically ill patients and might help to identify individuals at the highest risk for an adverse outcome.

The presence of fQRS is a common electrocardiographic finding in patients with STEMI undergoing pPCI, as well as other myocardial pathologies associated with myocardial scarring and delayed ventricular conduction. Although the underlying electrophysiological mechanisms are not completely resolved, heterogeneous activation resulting from delayed conduction within the scarred tissue appears to cause surface ECG patterns such as fQRS [22]. Depending on the definition and the ECG filter settings, prevalence ranges between 35 and 60% in a STEMI population as described by Lou et al. [23]. Furthermore, an increased risk for the occurrence of ventricular tachycardia, in-hospital major adverse cardiac events (MACE), and even mortality has already been demonstrated in these patients [24,25,26].

Currently, there exists no uniform definition for fQRS. Even though the presence of fQRS in a single lead might be clinically relevant, the classic definition with fQRS in ≥2 contiguous leads appears to have a higher positive predictive value for a negative clinical outcome, such as heart failure and mortality in a STEMI population [12]. Consequently, the definition suggested by Das et al. was used in this study, defining fQRS as the additional R-wave (R‘) or notching in the nadir of the S-wave or the presence of > 1 R in 2 contiguous leads corresponding to a major coronary artery territory on the resting 12-lead ECG [20]. Areas of uncertainty, such as wide QRS patterns in patients with conduction system disease or the distinction from artifacts, are to be addressed. To reduce intra- and inter-observer variability, machine learning algorithms for fQRS quantification have already been proposed [14]. These algorithms might allow the development and use of continuous fragmentation scores rather than binary models.

The association between fQRS and elevated CRP levels is pathophysiological plausible, given the well-established role of CRP as a marker of inflammation and myocardial injury in ischemia–reperfusion settings. Numerous studies have demonstrated that patients with STEMI exhibit significantly higher CRP levels compared to those with non–ST-elevation myocardial infarction (NSTEMI), highlighting the greater inflammatory burden associated with STEMI [27]. Moreover, the rate of change in CRP concentrations has been independently associated with 30-day mortality [28]. In STEMI patients, elevated CRP levels serve as a reliable predictor of both heart failure and mortality [29]. Admission CRP levels have also been shown to correlate strongly with angiographic indicators of disease severity, such as TIMI flow grade and SYNTAX score, particularly in patients undergoing pPCI [30,31]. Several interventional and pilot trials even address the use of IL-1 receptor antagonists to reduce inflammatory processes in STEMI patients [32]. Moreover, CRP apheresis appears to be a promising therapeutic target in patients with acute myocardial infarction, with ongoing larger randomized trials to evaluate the effect on hard clinical outcome parameters such as heart failure and mortality.

fQRS appears to be a clinically relevant parameter in other non-ischemic pathologies associated with inflammation and fibrosis of the myocardial tissue. Conditions such as arrhythmogenic cardiomyopathy (ACM), hypertrophic cardiomyopathy (HCM), and dilated (non-ischemic) cardiomyopathy have been identified to have higher prevalences of fQRS; especially in patients with HCM, these ECG changes were associated with a higher burden of ventricular arrhythmias and even cardiac death [26]. The clinical relevance of fQRS is further underscored by its presence in various systemic inflammatory conditions, including systemic lupus erythematosus (SLE), rheumatoid arthritis, and liver cirrhosis, as well as in iatrogenic clinical scenarios such as chest radiotherapy for malignancies. These findings suggest that fQRS may reflect subclinical myocardial injury or fibrosis induced by acute or chronic inflammation. Accordingly, fQRS may serve as an early, non-invasive electrocardiographic marker for detecting subclinical myocardial fibrosis in both cardiac and non-cardiac inflammatory disorders. Especially in the clinical context of STEMI, both fQRS and CRP are parameters easy to evaluate and might be even more useful than complex scoring systems or algorithms to identify patients at the highest risk for an adverse outcome. While this trial is only hypothesis-generating, these speculations should be applied with caution. Further investigations are necessary to link a high inflammatory burden to the occurrence of fQRS.

## 5. Strengths and Limitations

A key strength of this study is the homogeneity of the patient population, comprising 122 STEMI patients selected from a cohort of 179 individuals. By excluding subjects with clinical conditions that could potentially confound the analysis—such as active infections, permanent pacemakers, or other factors known to alter CRP levels or QRS morphology—the resulting findings are likely to be robust and internally valid. Furthermore, the use of multivariable regression analysis in this study enables the identification of true independent predictors, thereby strengthening the validity of the observed associations. One of the main limitations of this study is the relatively small sample size. As a result, the limited number of patients within each subgroup may have reduced the statistical power to detect potentially significant associations for certain variables. The single-center study design is inherently associated with certain limitations, such as regional differences in the healthcare system, as well as the representation of specific ethnical groups within the patient population. In this population, 7 patients (5.7%) were treated with thrombolysis. These percentages depend on the availability of primary or rescue PCI and might vary among different medical systems and geographical regions. Due to the given sample size, the study exhibits limited statistical power. Although the required sample size for the study was not determined a priori, a post hoc power analysis was conducted based on the results of the logistic regression, showing 81% power with the logit modeling obtained results. Consequently, the results and their interpretation cannot be extrapolated to the general population beyond this study. Finally, CRP only serves as an unspecific laboratory indicator for inflammation, while pathophysiological aspects of inflammation or fQRS were not evaluated in this trial.

## 6. Conclusions

In this study, a correlation between fQRS and CRP levels in patients with STEMI undergoing pPCI could be demonstrated. The analysis of fQRS might add additional value for the evaluation of patients presenting with myocardial ischemia. However, the clinical relevance as well as the association to extensive inflammation still requires confirmation in larger multi-center studies.

## Figures and Tables

**Table 1:** Baseline characteristics of STEMI patients.

PARAMETER	All, M (Q1–Q3)	with _f_QRS, M (Q1–Q3)	Without _f_QRS, M (Q1–Q3)	*p*
n	122	41	81	
Men (%)	83 (68.0)	33 (80.5)	50 (61.7)	**0.036**
Women (%)	39 (32.0)	8 (19.5)	31 (38.3)	**0.036**
Age, years	63 (56–70)	62 (57–69)	63 (56–71)	0.952
Height, cm	170 (166–174)	170 (168–176)	170 (165–173)	0.093
Weight, kg	78 (71.3–88.0)	78 (72–86)	79 (71–88)	0.92
BMI, kg/m^2^	26.9 (24.3–30.4)	27.0 (24.0–29.1)	26.8 (24.4–30.8)	0.318
Pulse, beat/min	74 (65–85)	74 (66–80)	72 (65–86)	0.899
SBP, mm Hg	130 (111–140)	130 (100–140)	130 (119–140)	0.344
DBP, mm Hg	80 (70–90)	80 (64–86)	80 (70–90)	0.259
Glucose, mmol/L	6 (4.6–7.7)	5.1 (4.2–7.1)	6.3 (4.9–9.2)	0.894
CRP, mg/L	1 (0.8–1.2)	0.9 (0.8–1.1)	1.1 (1.0–1.3)	0.024
Creatinine, mg/dL	87.5 (76.0–108.5)	85.5 (75.8–98.5)	89 (76–128)	0.030
GFR, mL/min/m^2^	60 (47.3–73.3)	60.5 (50.8–71.8)	60.0 (40.0–74.0)	0.049
Urea, mmol/L	6.0 (4.6–7.7)	5.1 (4.2–7.1)	5.5 (4.9–9.2)	0.020
ASAT, mmol/L	70.0 (44.0–120.0)	68 (44–116)	84 (56–144)	0.281
ALAT, mmol/L	42 (28.0–61.0)	44.5 (29.0–56.5)	38 (28.0–85.3)	0.243
Troponin, ng/mL	1200 (568–2657)	1200 (478–2696)	993 (699–2525)	0.202
LDH, mmol/L	619.5 (538.3–1026.8)	596.0 (518.5–916.3)	769.0 (567.0–1200.0)	0.249

**Table 2:** Comorbidities of STEMI patients.

PARAMETER	All	with _f_QRS	Without _f_QRS	*p*
DM2, n (%)	31 (25.4)	10 (24.4)	21 (25.92)	0.854
CKD, n (%)	22 (18.0)	9 (22.0)	13 (16.05)	0.424
AH, n (%)	119 (97.5)	40 (97.6)	78 (96.30)	0.711
Prior Stroke, n (%)	10 (8.2)	4 (9.8)	6 (7.41)	0.656
Dyslipidaemia, n (%)	122 (100)	41 (100)	80 (100)	0.475
AF, n (%)	14 (11.5)	4 (9.8)	9 (11.1)	0.819
Prior MI, n (%)	20 (16.4)	7 (17.1)	13 (16.1)	0.886

**Table 3:** Echocardiographic parameters of STEMI patients.

PARAMETER	All, M (Q1–Q3)	with fQRS, M (Q1–Q3)	Without fQRS, M (Q1–Q3)	*p*
LVEF, %	54 (48.3–59.8)	55 (48.0–58.0)	54.5 (49.8–60.0)	0.332
FS EF %	28 (25–32)	29 (24–32)	28 (25–32)	0.478
EDV, mm	4.8 (4.6–5.1)	5.1 (4.6–5.1)	4.8 (4.6–5.0)	0.369
ESV, mm	3.5 (3.2–3.7)	3.5 (3.3–4.0)	3.5 (3.2–3.7)	0.138
IVS, mm	1.1 (1.0–1.2)	1.1 (1.0–1. 2)	1.2 (1.0–1.2)	0.185
Pulmonary pressure, m	35 (28–45)	32 (29–41)	35 (28–47)	0.645

**Table 4:** Procedural angiographic parameters of STEMI patients.

PARAMETER	Whole Cohort	with fQRS	Without fQRS	*p*
Thrombolysis, n (%)	7 (5.7)	2 (3.7)	5 (6.2)	0.772
CAG	120 (98.4)	39 (95.1)	80 (98.8)	0.220
LAD, n (%)	79 (64.8)	26 (63.4)	52 (64.2)	0.933
RCA, n (%)	47 (38.5)	16 (39.0)	31 (38.3)	0.936
CX, n (%)	14 (11.5)	3 (7.3)	11 (13.6)	0.306
Stenting, n (%)	83 (68.0)	27 (65.9)	55 (67.9)	0.820
Onset-to-door, min		120 (60; 240)	60 (60; 1080)	0.082
Door-to-ballon, min		35 (30; 50)	30 (20; 40)	0.069
Anterior wall MI	41 (33.6%)	16 (39.0%)	25 (30.9%)	0.368
Posterior wall MI	81 (66.4%)	25 (61.0%)	56 (69.1%)	0.854
TIMI, 0	84 (68.9%)	31 (75.6%)	53 (65.4%)	0.654
1	35 (28.7%)	9 (21.9%)	26 (32.1%)	
2	2 (1.6%)	0	2 (2.5%)	
3	1 (0.8%)	1 (2.5%)	0	
Killip, I	85 (69.7%)	26 (63.4%)	59 (72.8%)	0.465
III	20 (16.4%)	9 (22.0%)	11 (13.6%)	
IV	17(13.9%)	6 (14.6%)	11(13.6%)	

**Table 5:** Univariable regression analysis of fQRS risk factors of STEMI patients.

Variable	Coefficient ± SE	*p*-Level
Glucose	−0.0015 ± 0.0033	0.655
NT-proBNP	0.0062 ± 0.0047	0.195
CRP	1.4190 ± 0.5794	0.014 *
Creatinine	0.0088 ± 0.0047	0.058
GFR	−0.0182 ± 0.0091	0.050 *
Urea	−0.0014 ± 0.0032	0.654
ASAT	0.0013 ± 0.0015	0.400
Onset-to-door time (min)	0.0031 ± 0.0015	0.035 *
Door-ballon time (min)	0.0165 ± 0.0088	0.061
MI location	0.3602 ± 0.4004	0.368
TIMI	1 −0.6012 ± 0.4651	0.203
	2 −15.02 ± 1455.40	0.99
	3 16.11 ± 1455.40	0.99
Killip	III 0.2007 ± 0.45	0.655
	III 0.6188 ± 0.50	0.223
	IV 0.2133 ± 0.56	0.703

*—coefficient of model statistically different from 0 at *p* < 0.005.

**Table 6:** Multivariable regression analysis of fQRS risk factors of STEMI patients.

Variable	Coefficient ± SE	OR, CI95%	*p*-Level
Intercept	−1.5744 ± 0.9773	-	0.107
GFR	−0.0117 ± 0.0101	0.988 (0.978; 0.998)	0.249
CRP	1.1704 ± 0.5777	3.223 (1.809; 5.743)	0.043 *
Onset-Door time	1.0025 ± 0.0015	1.0025 (1.0009; 1.004)	0.106

*—coefficient of model statistically different from 0 at *p* < 0.005.

## Data Availability

The raw data supporting the conclusions of this article will be made available by the Corresponding author (zsnaufal@gmail.com) on request.

## Abbreviations

The following abbreviations are used in this manuscript:

ACS	acute coronary syndrome
ACM	arrhythmogenic cardiomyopathy
ASAT	aspartate aminotransferase
CA	coronary angiography
CVD	cardiovascular disease
ELISA	enzyme-linked immunosorbent assay
ESC	European Society of Cardiology
fQRS	QRS fragmentation
GCP	Good Clinical Practice
GFR	glomerular filtration rate
HCM	hypertrophic cardiomyopathy
H-FABP	Heart-type Fatty Acid Binding Protein
MACE	major adverse cardiac events
MI	myocardial infarction
NSTEMI	non-ST elevation myocardial infarction
PCI	percutaneous coronary intervention
pPCI	primary percutaneous coronary intervention
sST2	soluble ST2
SLE	systemic lupus erythematosus
STEMI	ST-segment elevation myocardial infarction

## References

[1] GBD 2017 Causes of Death Collaborators (2018). Global, regional, and national age-sex-specific mortality for 282 causes of death in 195 countries and territories, 1980–2017: A systematic analysis for the Global Burden of Disease Study 2017. Lancet.

[2] Foreman K.J., Marquez N., Dolgert A., Fukutaki K., Fullman N., McGaughey M., Pletcher M.A., Smith A.E., Tang K., Yuan C.W. (2018). Forecasting life expectancy, years of life lost, and all-cause and cause-specific mortality for 250 causes of death: Reference and alternative scenarios for 2016-40 for 195 countries and territories. Lancet.

[3] Timmis A., Vardas P., Townsend N., Torbica A., Katus H., De Smedt D., Gale C.P., Maggioni A.P., Petersen S.E., Huculeci R. (2022). European Society of Cardiology: Cardiovascular disease statistics 2021: Executive Summary. Eur. Heart J. Qual. Care Clin. Outcomes.

[4] Byrne R.A., Rossello X., Coughlan J.J., Barbato E., Berry C., Chieffo A., Claeys M.J., Dan G.A., Dweck M.R., Galbraith M. (2023). 2023 ESC Guidelines for the management of acute coronary syndromes. Eur. Heart J..

[5] Zagidullin N., Motloch L.J., Gareeva D., Hamitova A., Lakman I., Krioni I., Popov D., Zulkarneev R., Paar V., Kopp K. (2020). Combining Novel Biomarkers for Risk Stratification of Two-Year Cardiovascular Mortality in Patients with ST-Elevation Myocardial Infarction. J. Clin. Med..

[6] Xia W., Feng X.Y. (2018). Fragmented QRS (fQRS) Complex Predicts Adverse Cardiac Events of ST-Segment Elevation Myocardial Infarction Patients Undergoing Percutaneous Coronary Intervention and Thrombolysis. Med. Sci. Monit..

[7] Tanriverdi Z., Colluoglu T., Unal B., Dursun H., Kaya D. (2018). The prognostic value of the combined use of QRS distortion and fragmented QRS in patients with acute STEMI undergoing primary percutaneous coronary intervention. J. Electrocardiol..

[8] Yildirim E., Karacimen D., Ozcan K.S., Osmonov D., Turkkan C., Altay S., Ceylan U.S., Ugur M., Bozbay M., Erdinler I. (2014). The relationship between fragmentation on electrocardiography and in-hospital prognosis of patients with acute myocardial infarction. Med. Sci. Monit..

[9] Ma L., Ma S., Lv J., Zhang Y., Liu Z., Bu P., Li Z., Wang L. (2016). Fragmented QRS complex on ECG is associated with ventricular arrhythmias in patients with a prior myocardial infarction. Acta Cardiol..

[10] Redfors B., Kosmidou I., Crowley A., Maehara A., Ben-Yehuda O., Arif A., Dizon J.M., Mintz G.S., Stone G.W. (2018). Prognostic significance of QRS fragmentation and correlation with infarct size in patients with anterior ST-segment elevation myocardial infarction treated with percutaneous coronary intervention: Insights from the INFUSE-AMI trial. Int. J. Cardiol..

[11] Kanitsoraphan C., Rattanawong P., Mekraksakit P., Chongsathidkiet P., Riangwiwat T., Kanjanahattakij N., Vutthikraivit W., Klomjit S., Thavaraputta S. (2019). Baseline fragmented QRS is associated with increased all-cause mortality in heart failure with reduced ejection fraction: A systematic review and meta-analysis. Ann. Noninvasive Electrocardiol..

[12] Tanriverdi Z., Dursun H., Colluoglu T., Kaya D. (2017). Single Derivation Fragmented QRS Can Predict Poor Prognosis in Successfully Revascularized Acute STEMI Patients. Arq. Bras. Cardiol..

[13] Umapathy S., Yadav R., Goswami K.C., Karthikeyan G., Parakh N., Bahl V.K. (2018). Prognostic significance of fragmented QRS in patients with ST-elevation myocardial infarction undergoing revascularization. Indian Heart J..

[14] Villa A., Vandenberk B., Kentta T., Ingelaere S., Huikuri H.V., Zabel M., Friede T., Sticherling C., Tuinenburg A., Malik M. (2022). A machine learning algorithm for electrocardiographic fQRS quantification validated on multi-center data. Sci. Rep..

[15] Kurtul A., Duran M. (2017). Fragmented QRS complex predicts contrast-induced nephropathy and in-hospital mortality after primary percutaneous coronary intervention in patients with ST-segment elevation myocardial infarction. Clin. Cardiol..

[16] Kadi H., Inanir A., Habiboglu A., Ceyhan K., Koc F., Celik A., Onalan O., Arslan S. (2012). Frequency of fragmented QRS on ECG is increased in patients with rheumatoid arthritis without cardiovascular disease: A pilot study. Mod. Rheumatol..

[17] Erdem F.H., Tavil Y., Yazici H., Aygul N., Abaci A., Boyaci B. (2013). Association of fragmented QRS complex with myocardial reperfusion in acute ST-elevated myocardial infarction. Ann. Noninvasive Electrocardiol..

[18] Ibanez B., James S., Agewall S., Antunes M.J., Bucciarelli-Ducci C., Bueno H., Caforio A.L.P., Crea F., Goudevenos J.A., Halvorsen S. (2018). 2017 ESC Guidelines for the management of acute myocardial infarction in patients presenting with ST-segment elevation: The Task Force for the management of acute myocardial infarction in patients presenting with ST-segment elevation of the European Society of Cardiology (ESC). Eur. Heart J..

[19] Thygesen K., Alpert J.S., Jaffe A.S., Chaitman B.R., Bax J.J., Morrow D.A., White H.D., Executive Group on Behalf of the Joint European Society of Cardiology/American College of Cardiology/American Heart Association/World Heart Federation Task Force for the Universal Definition of Myocardial Infarction (2018). Fourth Universal Definition of Myocardial Infarction. Circulation.

[20] Das M.K., Khan B., Jacob S., Kumar A., Mahenthiran J. (2006). Significance of a fragmented QRS complex versus a Q wave in patients with coronary artery disease. Circulation.

[21] Peduzzi P., Concato J., Kemper E., Holford T.R., Feinstein A.R. (1996). A simulation study of the number of events per variable in logistic regression analysis. J. Clin. Epidemiol..

[22] Das M.K., Saha C., El Masry H., Peng J., Dandamudi G., Mahenthiran J., McHenry P., Zipes D.P. (2007). Fragmented QRS on a 12-lead ECG: A predictor of mortality and cardiac events in patients with coronary artery disease. Heart Rhythm..

[23] Luo G., Li Q., Duan J., Peng Y., Zhang Z. (2020). The Predictive Value of Fragmented QRS for Cardiovascular Events in Acute Myocardial Infarction: A Systematic Review and Meta-Analysis. Front. Physiol..

[24] Kanjanahattakij N., Rattanawong P., Riangwiwat T., Prasitlumkum N., Limpruttidham N., Chongsathidkiet P., Vutthikraivit W., Crossey E. (2018). Fragmented QRS and mortality in patients undergoing percutaneous intervention for ST-elevation myocardial infarction: Systematic review and meta-analysis. Ann. Noninvasive Electrocardiol..

[25] Turkmen S., Bozkurt M., Hosoglu Y., Gol M. (2024). Significance of fragmented QRS and predictors of outcome in ST-elevation myocardial infarction. J. Res. Med. Sci..

[26] Haukilahti M.A., Eranti A., Kentta T., Huikuri H.V. (2016). QRS Fragmentation Patterns Representing Myocardial Scar Need to Be Separated from Benign Normal Variants: Hypotheses and Proposal for Morphology Based Classification. Front. Physiol..

[27] Habib S.S., Kurdi M.I., Al Aseri Z., Suriya M.O. (2011). CRP levels are higher in patients with ST elevation than non-ST elevation acute coronary syndrome. Arq. Bras. Cardiol..

[28] Milwidsky A., Ziv-Baran T., Letourneau-Shesaf S., Keren G., Taieb P., Berliner S., Shacham Y. (2017). CRP velocity and short-term mortality in ST segment elevation myocardial infarction. Biomarkers.

[29] Stumpf C., Sheriff A., Zimmermann S., Schaefauer L., Schlundt C., Raaz D., Garlichs C.D., Achenbach S. (2017). C-reactive protein levels predict systolic heart failure and outcome in patients with first ST-elevation myocardial infarction treated with coronary angioplasty. Arch. Med. Sci..

[30] Tanveer S., Banu S., Jabir N.R., Khan M.S., Ashraf G.M., Manjunath N.C., Tabrez S. (2016). Clinical and angiographic correlation of high-sensitivity C-reactive protein with acute ST elevation myocardial infarction. Exp. Ther. Med..

[31] Karadeniz M., Duran M., Akyel A., Yarlioglues M., Ocek A.H., Celik I.E., Kilic A., Yalcin A.A., Ergun G., Murat S.N. (2015). High Sensitive CRP Level Is Associated with Intermediate and High Syntax Score in Patients with Acute Coronary Syndrome. Int. Heart J..

[32] Abbate A., Wohlford G.F., Del Buono M.G., Chiabrando J.G., Markley R., Turlington J., Kadariya D., Trankle C.R., Biondi-Zoccai G., Lipinski M.J. (2022). Interleukin-1 blockade with anakinra and heart failure following ST-segment elevation myocardial infarction: Results from a pooled analysis of the VCUART clinical trials. Eur. Heart J. Cardiovasc. Pharmacother..

